# The risk factors of type 2 diabetes in hypertensive subjects

**DOI:** 10.3389/fendo.2022.901614

**Published:** 2022-07-22

**Authors:** Yingqun Chen, Jiner Ma, Donghui Lu, Yefei Fang

**Affiliations:** ^1^ Department of General Practice, Yuyao People’s Hospital, Ningbo, China; ^2^ Department of Geriatrics, Yuyao People’s Hospital, Ningbo, China; ^3^ Emergency Trauma Department, Yuyao People’s Hospital, Ningbo, China

**Keywords:** hypertension, type 2 diabetes, diastolic blood pressure, creatinine, case-control

## Abstract

**Objective:**

Hypertension (HTN) and type 2 diabetes (T2DM) share common risk factors and usually co-occur. This study examined the relationship between HTN history and T2DM incidence in a cohort of Chinese hypertensive subjects.

**Methods:**

We recruited 443 cases (T2DM and HTN) and 443 sex- and age-matched controls (HTN). The history of peak systolic blood pressure (SBP) was divided into 140-159, 160-179, and ≥ 180 mmHg, and that of peak diastolic blood pressure (DBP) was divided into 90-99, 100-109, and ≥ 110 mmHg. Multiple binary logistic regression models were used to explore the association between controlled HTN status and T2DM.

**Results:**

Creatinine concentrations were higher in the cases than in the controls (*P* < 0.05). The HTN duration was longer in the cases than in the controls (14.7 years *vs.* 13.2 years; *P* < 0.05). Significant differences were also found in the history of peak SBP and DBP between the cases and controls (both *P* < 0.05). Creatinine, HTN duration, and family history of T2DM were risk factors for T2DM in hypertensive subjects, with odds ratios (95% confidence intervals) of 1.013 (1.004-1.022), 1.025 (1.003-1.047), and 5.119 (3.266-8.026), respectively. Compared with the lowest level of peak DBP, the odds ratio for T2DM at the highest level of peak DBP was 1.757 (1.074-2.969). Subgroups analyses showed that the effect of the history of peak DBP on T2DM was significantly modified by sex (*P*-interaction = 0.037).

**Conclusion:**

The highest DBP and the longest HTN duration were both independently associated with T2DM in hypertensive subjects.

## Introduction

Type 2 diabetes mellitus (T2DM) is a serious public health problem worldwide due to its considerable impact on human health and quality of life. Globally, approximately 463 million individuals had T2DM in 2019, accounting for 9.3% of the world’s population (1). The prevalence of diabetes has increased rapidly, and has been predicted to further increase from 6,059/100,000 cases in 2017 to 7,059 per 100,000 people in 2030 (2). Owing to its large population, China has the largest number of T2DM cases at 114 million (2).

Several longitudinal studies have indicated that hypertension (HTN) is an important risk factor for T2DM and can play a vital role in its prognosis (3–6). HTN and T2DM share common risk factors and usually co-occur. Approximately 58% of T2DM patients also have HTN (7). The influence of high blood pressure on impaired glucose metabolism is believed to be mainly exerted *via* altered endothelial permeability and oxidative stress, which in turn promote insulin resistance and pancreatic β-cell dysfunction (8–10). In addition, the extent of glucose metabolism impairment due to HTN may be governed by the history of peak high blood pressure and the duration of HTN. However, no study has yet focused on the relationship between the history of HTN (i.e., the history of peak high systolic blood pressure [SBP] and diastolic blood pressure [DBP] and the duration of HTN) and the incidence of T2DM. Elucidating this relationship will provide insights into the T2DM susceptibility of hypertensive patients and can inform preventive measures, especially in China, where there is a large number of both HTN and diabetes cases.

Therefore, we conducted a community-based case–control study to examine whether the history of HTN influences the incidence of T2DM in a cohort of Chinese hypertensive subjects.

## Materials and methods

### Study design

This was a population-based retrospective study with 1:1 sex- and age-matched case and control groups. Individuals with HTN alone and with both HTN and T2DM were recruited from the Department of Geriatrics in Yuyao People’s Hospital, Zhejiang, China. In total, 443 cases (with both T2DM and HTN) and 443 controls (with only HTN) from the same community and matched by sex and age (within 3 years) were included in the study. Subjects who were pregnant and those with secondary HTN, serious liver or kidney failure, psychosis, cancer, Alzheimer’s disease, type 1 diabetes, or a history of ischemic stroke or coronary heart disease were excluded. All participants provided written informed consent for inclusion in this study, and the Ethical Committee of Yuyao People’s Hospital approved the study.

Hypertensive subjects were diagnosed in hospitals according to the following criteria: (i) an SBP ≥ 140 mmHg and/or a DBP ≥ 90 mmHg, based on the mean of three measurements, and (ii) the use of antihypertensive medication. Diabetes was diagnosed according to the American Diabetes Association 2010 criteria (11) when the fasting plasma glucose concentration and the 2-h post-prandial plasma glucose concentration were higher than 7.0 mmol/L and 11.1 mmol/L, respectively, or the glycated hemoglobin concentration was higher than 6.5%.

### Data collection

A standard structured questionnaire was used to collect information on demographic and behavioral risk factors, including age, sex, smoking, alcohol consumption, sleeping duration, family history of HTN and T2DM, antihypertensive medication use, and HTN duration. Face-to-face interviews were conducted by trained physicians. During these interviews, the history of peak SBP and DBP was reported by the subjects, while the current SBP and DBP were measured by the physicians using a standard mercury sphygmomanometer on the right arm of the seated participants after a 5-min rest in the sitting position. The current SBP was divided into <120 mmHg, 120-139 mmHg, 140-159 mmHg, and ≥ 160 mmHg, and the current DBP was divided into <80 mmHg, 80-89 mmHg, 90-99 mmHg, and ≥ 100 mmHg. The history of peak high SBP was divided into 140-159 mmHg, 160-179 mmHg, and ≥ 180 mmHg, and the history of peak high DBP was divided into 90-99 mmHg, 100-109 mmHg, and ≥ 110 mmHg. Body mass index (BMI) was calculated as body weight (kg)/height squared (m^2^).

Blood samples were obtained after overnight fasting. All samples were examined for concentrations of total cholesterol, serum triglyceride (TG), uric acid (UA), low-density lipoprotein cholesterol (LDL), serum creatinine, homocysteine, and glucose. All of these clinical laboratory parameters were determined using routine laboratory methods. Total cholesterol, TG, LDL, glucose, and homocysteine concentrations were measured with the automatic biochemical analyzer HITACH 7080 using standard enzymatic methods. The serum concentrations of UA and creatinine were determined quantitatively using the uricase method and the Jaffe reaction method, respectively.

### Statistical analysis

Continuous variables were summarized as means ± standard deviations. Student’s *t*-test was used to compare continuous variables between the case and control groups, and the chi-square test was used to compare categorical variables between the groups. Multiple binary logistic regression models were used to explore the association between controlled HTN status and T2DM incidence. The strength effect estimates were measured using odds ratios (ORs) with 95% confidence intervals (CIs). The effects of sex (men/women), smoking (yes/no), drinking (yes/no), and sleeping duration (≥8 h/<8 h) were assessed using stratified analyses. All *P* values were two-tailed and considered to be statistically significant at < 0.05. Statistical analysis was performed using IBM SPSS software, version 19.0 (SPSS Inc., Chicago, IL, USA).

## Results

### Baseline characteristics

A total of 886 participants—443 cases (T2DM and HTN) and 443 controls (HTN)—were recruited in this matched case–control study. **Table 1** shows the baseline characteristics of the subjects. No significant differences were found in age, sex, BMI, UA concentrations, homocysteine concentrations, smoking status, or alcohol consumption between the cases and controls. The total cholesterol and LDL concentrations were higher and the sleeping durations were longer in the controls than in the cases (all *P* < 0.05). The TG, creatinine, and glucose concentrations were higher and the family history of T2DM was longer in the cases than in the controls (all *P* < 0.05; **Table 1**).

**Table 1:** The baseline characteristics of cases (T2DM and hypertension) and controls (hypertension).

	Controls	Cases	*t*/*χ^2^*	*P* value
Age, y	61.3 ± 10.9	62.4 ± 10.9	-1.54	0.13
Sex (Male/Female)	237/206	237/206		
BMI, Kg/m^2^	24.3 ± 3.0	24.5 ± 2.8	-1.11	0.27
TC, mmol/L	5.2 ± 1.0	5.0 ± 1.1	2.48	0.01
TG, mmol/L	2.0 ± 1.2	2.3 ± 2.6	-2.53	0.01
UA, μmol/L	350.9 ± 98.6	349.7 ± 93.9	0.19	0.85
LDL, mmol/L	3.1 ± 0.8	2.9 ± 0.8	3.13	<0.01
Crea, μmol/L	79.9 ± 17.6	83.6 ± 24.3	-2.57	0.01
Hcy, μmol/L	15.4 ± 12.7	14.8 ± 7.2	0.93	0.35
FBG, mmol/L	5.6 ± 1.2	6.9 ± 2.1	-11.57	<0.01
Sleeping status (≥ 8h/<8h)	153/295	125/323	4.09	0.04
Smoking (Yes/No)	152/296	138/310	1.00	0.32
Drinking (Yes/No)	38/410	32/416	0.56	0.46
Family history of T2DM (Yes/No)	28/420	118/220	66.28	<0.01

TC, total cholesterol; (TG), serum triglyceride; (UA), uric acid; (LDL), low-density lipoprotein cholesterol; (Crea), serum creatinine; (Hcy), homocysteine; and (FBG), first blood glucose; hypertension, (T2DM), type 2 diabetes mellitus.

The HTN durations were longer in the cases than in the controls (14.7 years *vs.* 13.2 years) (*P* < 0.05; **Table 2**). There were no significant differences in the status of HTN drug use or current SBP and DBP between the cases and controls (all *P* > 0.05; **Table 2**), but significant differences were found in the history of peak high SBP and DBP between the two groups (both *P* < 0.05; **Table 2**).

**Table 2:** The hypertensive characteristics of cases (T2DM and hypertension) and controls (hypertension).

	Controls	Cases	*t/χ^2^*	*P* value
Hypertension duration	13.2±7.0	14.7±7.9	-3.10	<0.01
Hypertension drug				
Yes	375	374	0.01	0.93
No	73	74		
Current SBP				
<120 mmHg	61	63	0.12	0.99
120-139 mmHg	244	244		
140-159 mmHg	134	131		
≥160 mmHg	9	10		
Current DBP				
<80 mmHg	110	124	1.26	0.74
80-89 mmHg	231	225		
90-99 mmHg	97	89		
≥ 100 mmHg	10	10		
History highest SBP				
140-159 mmHg	211	174	6.44	0.04
160-179 mmHg	161	181		
≥ 180 mmHg	76	93		
History highest DBP				
90-99 mmHg	283	245	7.05	0.02
100-109 mmHg	118	139		
≥ 110 mmHg	47	64		

### Multiple binary logistic regression models

The results of multiple binary logistic regression models showed that the TG and creatinine concentrations, HTN duration, and family history of T2DM were all risk factors for T2DM in HTN (all *P* < 0.05, **Table 3**), with ORs (95% CIs) of 1.134 (1.032-1.246), 1.013 (1.004-1.022), 1.025 (1.003-1.047), and 5.119 (3.266-8.026), respectively. Analysis of the history of peak high DBP showed that compared with the low DBP category (90-99 mmHg), the ORs for T2DM in the medium (100-109 mmHg) and high (≥110 mmHg) DBP categories were 1.398 (1.044-1.971) and 1.757 (1.074-2.969), respectively. Furthermore, a linear trend was found in the association between increments in the history of peak high DBP and the risk of T2DM (*P*-trend = 0.023).

**Table 3:** Multiple logistic regression analysis of the risk of T2DM in hypertension subjects.

	β	SE	Wald	OR (95%CI)	*P* value	*P*-trend^*^
Constant	-1.599	0.897	3.177		0.075	
Age	0.006	0.008	0.525	1.006 (0.990-1.021)	0.469	
Sex	0.053	0.082	0.417	1.054 (0.898-1.238)	0.518	
BMI	0.025	0.026	0.945	1.025 (0.975-1.078)	0.331	
TC	-0.255	0.207	1.516	0.775 (0.516-1.163)	0.218	
TG	0.126	0.048	6.882	1.134 (1.032-1.246)	0.009	
LDL	-0.176	0.127	1.938	0.838 (0.654-1.078)	0.164	
Crea	0.013	0.005	8.234	1.013(1.004-1.022)	0.004	
UA	-0.001	0.001	2.348	0.999 (0.997-1.000)	0.125	
FBG	-0.012	0.009	1.754	0.988 (0.972-1.006)	0.185	
Sleeping time	0.099	0.065	2.357	1.104 (0.973-1.253)	0.125	
Drinking	-0.361	0.278	1.683	0.697 (0.404-1.202)	0.195	
Smoking	-0.056	0.159	0.123	0.946 (0.692-1.292)	0.726	
Hypertension duration	0.024	0.011	5.124	1.025 (1.003-1.047)	0.024	
Hypertension drug	-0.233	0.205	1.292	0.792 (0.530-1.184)	0.256	
Current SBP	0.059	0.131	0.204	1.061 (0.820-1.372)	0.651	
Current DBP	-0.157	0.125	1.590	0.854 (0.669-1.091)	0.207	
History highest SBP						0.685
140-159				1.000		
160-179	0.143	0.189	0.573	1.154 (0.797-1.670)	0.449	
≥ 180	0.065	0.270	0.059	1.068 (0.629-1.812)	0.809	
History highest DBP						0.023
90-99				1.000		
100-109	0.335	0.200	2.802	1.398 (1.044-1.971)	0.044	
≥ 110	0.563	0.301	3.503	1.757 (1.074-2.969)	0.031	
Family history of T2DM	1.633	0.229	50.679	5.119 (3.266-8.026)	<0.001	

(TC), total cholesterol; (TG), serum triglyceride; (UA), uric acid; (LDL), low-density lipoprotein cholesterol; (Crea), serum creatinine; (Hcy), homocysteine and (FBG), first blood glucose. ^*^P-trend, estimated using the category numbers (1, 2, and 3) as a continuous variable.

In subgroups analyses, the association between the history of peak high DBP and the incidence of T2DM was significantly modified by sex (*P*-interaction = 0.037). In the male subgroup, compared with the low DBP category, the ORs for T2DM in the medium and high categories of peak high DBP were 1.381 (1.014-1.758) and 1.670 (1.259-2.181), respectively (**Table 4**).

**Table 4:** Subgroup analysis of the risk of history highest DBP (mmHg) to T2DM.

	History highest DBP (mmHg)				
	90-99	100-109	≥ 110	*P*-trend^*^	*p-*interaction
Sex					
Men	1 (Reference)	1.381 (1.014-1.758)	1.670 (1.259-2.181)	0.035	0.037
Women	1 (Reference)	1.278 (0.828-1.973)	1.255 (0.702-1.808)	0.707	
Drinking					
Yes	1 (Reference)	1.343 (1.147-1.549)	1.384 (1.026-1.764)	0.572	0.559
No	1 (Reference)	1.303 (0.956-1.775)	1.279 (0.814-2.011)	0.103	
Smoking					
Yes	1 (Reference)	0.936 (0.401-2.185)	1.830 (0.844-3.550)	0.644	0.769
No	1 (Reference)	1.148 (0.766-1.720)	1.645 (0.867-3.122)	0.145	
Sleeping time					
≥8h	1 (Reference)	0.946 (0.508-1.763)	1.371 (0.919-2.045)	0.556	0.695
<8h	1 (Reference)	1.075 (1.003-1.147)	2.227 (1.317-3.406)	0.115	

^*^P-trend, estimated using the category numbers (1, 2, and 3) as a continuous variable.

ng the category numbers (1, 2, and 3) as a continuous variable.

## Discussion

This study explored the relationship between the history of HTN and the incidence of T2DM in a cohort of Chinese hypertensive subjects. After controlling for potential confounders, the history of peak high DBP and the duration of HTN were both independent risk factors for T2DM.

Studies have reported a strong association between HTN and the risk of T2DM. In particular, even normal-high SBP levels (130-139 mmHg) were shown to be significant predictors of T2DM in men (OR = 1.43) (3), after adjusting for BMI and other conventional T2DM risk factors. In a 10-year prospective study in Korea, subjects with baseline stage 2 HTN (≥ 160/100 mmHg) were at a 1.60-fold higher risk of developing diabetes than normal subjects (12). In a recent 6-year rural Chinese cohort study, restricted cubic spline analysis revealed an increased risk of T2DM with increasing mean arterial pressure among women (13). Consistent with previous studies, we discovered that the history of peak high DBP and duration of HTN were associated with T2DM in hypertensive subjects. This association remained after controlling for HTN drug use and current SBP and DBP, suggesting that the association between the history of long-term high blood pressure and the incidence of T2DM is independent of the association between the duration of HTN and the incidence of T2DM.

The direct causal link between the pathophysiological mechanisms of HTN and T2DM has not yet been completely identified, but several hypotheses have been proposed. HTN has been shown to induce microvascular changes (14) that result in insulin resistance *via* impaired glucose and nutrient delivery to skeletal muscle, which may in turn contribute to diabetes. Endothelial dysfunction is also related to insulin resistance and may partially account for the strong association between HTN and T2DM (15, 16). Inflammatory markers and oxidative stress also play vital roles in both HTN and diabetes (9, 17). C-reactive protein and interleukin 6 may contribute to the incidence of T2DM *via* interaction with the insulin signaling pathway and pancreatic β-cell function (18). Oxidative stress, which plays a critical role in pancreatic β-cell dysfunction, is also associated with high blood pressure (9).

In this study, the serum TG concentration and a family history of T2DM were found to be common risk factors for T2DM. In addition, the serum creatinine concentration was shown to be a potential risk factor for T2DM in hypertensive subjects. A recent dose–response meta-analysis of six cohort studies showed a negative association between the serum creatinine concentration and T2DM risk (19); the risk was increased by 7% with each 0.1 mg/dL decrease in the serum creatinine concentration. Serum creatinine is the only metabolite of creatine in skeletal muscle and represents the level of skeletal muscle mass. Low skeletal muscle mass can lead to insulin resistance (20, 21) and reduced glucose uptake by skeletal muscle, thereby resulting in the development of T2DM. In contrast to the finding of the previous meta-analysis, we observed a positive association between the serum creatinine concentration and T2DM risk in hypertensive subjects. The reason for this finding is not clear, but we hypothesize that the expression of creatine kinase in hypertensive patients is abnormal (22, 23). High creatine kinase activity promotes HTN through enhanced vascular contractility and sodium retention in the kidneys. Creatine kinase is tightly bound near ATPases and catalyzes the conversion of creatine to creatinine in serum (22). The specific physiological mechanisms need to be investigated in future studies.

To the best of our knowledge, this is the first study to explore the potential association between the history of peak high blood pressure and the incidence of T2DM in a cohort of Chinese hypertensive subjects. However, this study has a few limitations. First, the history of peak blood pressure was self-reported by the participants. Therefore, to reduce potential recall bias, we classified the blood pressure values into groups instead of using the specific blood pressure values. Second, the study sample included only Han Chinese adults, and thus, the findings cannot be generalized to other ethnicities in China or populations in other countries. Caution should be exercised when extrapolating our results to other populations. Finally, owing to the limitations inherent in case–control studies, longer follow-up cohort studies and intervention trials are needed to confirm our findings.

## Conclusions

This study demonstrated that both the history of peak high DBP and the duration of HTN were independently associated with the incidence of T2DM in hypertensive subjects. This suggests that diabetes prevention measures should target hypertensive subjects with extremely high DBP and longer HTN duration as they are at a high risk of developing diabetes. Our findings should be verified by a longitudinal study in which a cohort of cases with HTN and a cohort of control subjects are followed up to track the development of T2DM.

## Data availability statement

The original contributions presented in the study are included in the article/supplementary material. Further inquiries can be directed to the corresponding author.

## Ethics statement

The studies involving human participants were reviewed and approved by yuyao People’s Hospital. The patients/participants provided their written informed consent to participate in this study.

## Author contributions

All authors listed have made a substantial, direct, and intellectual contribution to the work and approved it for publication.

## Conflict of interest

The authors declare that the research was conducted in the absence of any commercial or financial relationships that could be construed as a potential conflict of interest.

## Publisher’s note

All claims expressed in this article are solely those of the authors and do not necessarily represent those of their affiliated organizations, or those of the publisher, the editors and the reviewers. Any product that may be evaluated in this article, or claim that may be made by its manufacturer, is not guaranteed or endorsed by the publisher.
